# Autistic Symptoms in Schizophrenia: Impact on Internalized Stigma, Well-Being, Clinical and Functional Characteristics

**DOI:** 10.3389/fpsyt.2022.801651

**Published:** 2022-03-30

**Authors:** Stefano Barlati, Gabriele Nibbio, Donato Morena, Paolo Cacciani, Paola Corsini, Alessandra Mosca, Giacomo Deste, Vivian Accardo, Valentina Regina, Jacopo Lisoni, Cesare Turrina, Paolo Valsecchi, Antonio Vita

**Affiliations:** ^1^Department of Mental Health and Addiction Services, ASST Spedali Civili of Brescia, Brescia, Italy; ^2^Department of Clinical and Experimental Sciences, University of Brescia, Brescia, Italy; ^3^Department of Mental Health, Salerno, Italy; ^4^Department of Molecular and Translational Medicine, University of Brescia, Brescia, Italy

**Keywords:** schizophrenia, self-stigma, functioning, stigma, Autism Spectrum Disorder, well-being, internalized stigma

## Abstract

Autism Spectrum Disorders (ASD) symptoms and internalized stigma (or self-stigma) can have a negative impact on cognitive and functional outcomes in people living with schizophrenia. Aim of the present study were to assess and compare internalized stigma, subjective well-being and other socio-demographic, clinical and functional characteristics in people diagnosed with schizophrenia with and without prominent autistic features. Ninety-four inpatients were assessed with measures of internalized stigma, subjective well-being, global clinical severity, schizophrenia symptoms severity, real-world functioning, medication side effects and attitude toward prescribed medications. Subjects with high levels of ASD symptoms were identified with the PANSS Autism Severity Score and compared to other participants. Predictors of prominent ASD features were also assessed. Thirteen patients showed prominent ASD symptoms. They were characterized by fewer years of education, worse real-world functioning and greater symptoms severity. No between-group differences were observed regarding subjective well-being and global internalized stigma severity; however, participants in the “autistic schizophrenia” group showed better stigma resistance. A worse clinical condition and fewer years of education emerged as predictors of autistic schizophrenia. Despite showing a more severe clinical presentation of the disorder and worse functional impairment, participants with prominent ASD symptoms do not present worse subjective well-being or more severe internalized stigma; on the contrary, they show better stigma resistance. ASD symptoms could therefore play a protective role in the internalization of stigma.

## Introduction

### Background

Autism Spectrum Disorders (ASD) and schizophrenia are considered separate entities in current nosological classifications (1); however, autistic traits represent one of the core features of schizophrenia, and have been described as a central element of the disorder since its earliest conceptualizations (2).

In fact, while ASD and schizophrenia emerge at different developmental periods and have clearly distinctive characteristics, a growing body of scientific literature is focusing on the overlaps between the two spectra, highlighting important similarities in clinical, cognitive and neurobiological aspects (3–10). ASD features are a frequent finding in people living with schizophrenia (11–13); while they share similarities and overlaps with negative symptoms, which represent one of the core aspects of schizophrenia (14–16), they are also characterized by a wide range of clinical, cognitive and functional correlates. Recent evidence suggests that ASD symptoms are correlated with more severe deficits in social cognition and greater impairment in real-world functioning (17–21) and also with greater impairments in the ability to judge the quality of everyday functioning and well-being (22). In fact, according to a recent network analysis, ASD features appear to be negatively and more closely related to social functioning than psychotic symptoms in people diagnosed with psychotic disorders or with familiar risk for psychosis (23). ASD features also appear to be associated with poorer response to antipsychotic treatment (24, 25) and to a particular coping profile (26). A recent study, conducted on a large sample of people living with schizophrenia, found that participants with prominent ASD symptoms show poorer performance on most neurocognitive domains and in social cognition, as well as poorer functional capacity, real-world interpersonal relationships and participation in community-living activities, but better social acceptability (27).

However, some studies also report that ASD features may have a protective role on social cognition and on functioning (28), which could depend on the severity of psychotic symptoms (29): in this regard, the impact of ASD symptoms on cognitive and functional outcomes of schizophrenia represents a topic of valuable scientific discussion, with important clinical repercussions that remain to be further explored.

People living with schizophrenia are also at high risk of suffering stigma, which can be defined as the co-occurrence of labeling, stereotyping, separation, status loss, and discrimination, and incorporating it into their personal value system and sense of self (30–34).

Internalized stigma (also known as self-stigma) has an important impact on psychosocial functioning and on many recovery-related outcomes: it is consistently associated with more severe depressive and anxiety symptoms, lower quality of life and diminished self-esteem, poorer social and vocational functioning, lesser support and lower treatment adherence (35–37). It also represents a significant mediator between avolition and resilience (38) and between insight and depressive symptoms (39) and can affect identity changes in individuals with psychotic disorders (40).

While ASD symptoms and internalized stigma both represent important aspects of schizophrenia, with relevant repercussions on clinical, functional and rehabilitation outcomes, the potential correlations between these two elements are still scarcely investigated.

To date, to the best of our knowledge, only one study, conducted on a sample of 127 subjects diagnosed with schizophrenia spectrum disorders, has explored the potential associations between self-reported ASD features and internalized stigma, finding that more severe ASD features appear to represent an individual predictor of worse internalized stigma (41).

Moreover, no assessment of internalized stigma and of subjective well-being has been previously conducted in individuals living with schizophrenia showing prominent ASD features. Considering that these individuals may represent a sub-population that appears to be characterized by specific clinical, cognitive and functional correlates (27, 42), better understanding the severity of internalized stigma and of it components as well as the level of subjective well-being in these patients could be an important step in order to develop and implement personalized and targeted interventions (15).

### Aims

The aims of the present study were to assess internalized stigma, subjective well-being and real-world functioning, as well as other relevant socio-demographic, clinical and functional characteristics, in people living with schizophrenia, comparing participants with and without prominent ASD symptoms. The main hypothesis of the present study is that individuals with high levels ASD symptoms may present differences in the severity of internalized stigma or of its components, as well as other distinctive clinical and functional characteristics.

## Materials and Methods

### Sample

All patients with a diagnosis of schizophrenia accessing the residential and rehabilitative centers of the Department of Mental Health and Addiction Services of the Spedali Civili Hospital of Brescia, Italy, from January 2019 to December 2019 were invited to participate in this cross-sectional observational study by their treating physician.

Residential and rehabilitation centers of the Spedali Civili Hospital in Brescia are open inpatient structures receiving funding by the National Health System. Psychiatric and psychosocial treatment are provided to all admitted patients. The rehabilitation program is composed of psychiatric case management, pharmacological treatment, with nursing staff tasked to administer medications, and socialization and leisure activities. Evidence-based psychosocial interventions are also offered to all patients; at the time of assessment, however no participant had completed the treatment program. Further details on standard treatment provided in these centers are reported elsewhere (43).

To be included, patients had to have a clinical diagnosis of schizophrenia according to DSM-5 criteria (1) and a good knowledge of the Italian language. Moreover, antipsychotic treatment had to be stable, regarding both prescribed medications, doses and posology, for at least one month.

Patients younger than 18 or with a history of neurologic disorder or of substance abuse were excluded. Evidence of clinical instability in the month before assessment, including either admission in a psychiatric ward, change in type or dose of antipsychotic medication, increased frequency of contact with mental health services, indications of clinical instability reported by relatives, caregivers, or clinical team also represented a reason for exclusion.

Consent to participate in the study was provided through a written and signed form. The study has been carried out in accordance with the Code of Ethics of the World Medical Association and the Declaration of Helsinki. The Ethical Committee of Brescia approved the study with the Project Identification Code NP 2902. All precautions were taken for the management of sensitive data and no monetary compensation was provided to participants for their inclusion in the study.

### Assessment

Assessment was carried out in a single visit, during which participants completed self-rated measures and a trained physician, independent from the standard care process, administered all the investigator-rated measures. Raters were trained and certified for the administration of psychometric scales and showed good inter-rater reliability, assessed in previous studies conducted in the same Department (44). Socio-demographic and medication-related variables were collected from the rehabilitation center clinical records. Antipsychotic medication dose and total medication dose was calculated using Defined Daily Dose (DDD) methodology, as recommended by the WHO (45).

### ASD Symptoms Severity

The PANSS Autism Severity Score (PAUSS) (46) was used to assess the severity of ASD symptoms. The PAUSS is a scale derived from the Positive and Negative Syndrome Scale (PANSS) (47) and is obtained by summing the score of the items N1 (“blunted affect”), N3 (“poor rapport”), N4 (“social withdrawal”), N5 (“difficulties in abstract thinking”), N6 (“lack of spontaneity and flow of conversation”), N7 (“stereotyped thinking”), G5 (“mannerism”), and G15 (“preoccupation”). It is designed specifically to assess ASD symptoms severity in people diagnosed with schizophrenia spectrum disorders in clinical settings and its validity and accuracy are comparable to those of more elaborate and time-consuming instruments (48), such as the Autism Diagnostic Observation Schedule (ADOS) (49) and the Autism Diagnostic Interview-Revised (ADI-R) (50). Its score ranges from 8 to 56, with higher scores indicating more severe ASD symptoms.

On the basis of the cut-off scores identified in the original validation study (46), the sample was divided in two sub-groups: participants showing prominent ASD symptoms, or with “autistic schizophrenia” (PAUSS score ≥ 30), and participants with intermediate or low levels of ASD symptoms, or without “autistic schizophrenia” (PAUSS score <30).

### Clinical and Functional Measures

The severity of schizophrenia symptoms was assessed independently from the severity of ASD symptoms using the remaining items of the PANSS (PANSSminusPAUSS). This approach has been adopted and validated in various previous studies, conducted on large samples of patients (27, 51), in order to avoid collinearity between the PAUSS and the PANSS total score.

Global clinical situation was assessed with the Clinical Global Impression-Severity scale (CGI-S) (52) in order to provide a brief and comprehensive measure of illness severity. The score ranges from 1 to 7, with higher scores indicating more severe psychopathology.

Internalized stigma was measured with the Internalized Stigma of Mental Illness (ISMI) (53), an instrument including 29 items, each self-rated on a four-point anchored Likert scale ranging from strongly disagree to strongly agree, that investigates the person's identity and experience with mental illness. The ISMI represents a well-validated and widely used measure of self-stigma with good psychometric proprieties (54). Higher scores correspond to worse internalized stigma experience.

According to a recent factor analysis (55), three different factors compose the ISMI total score: Experiential Stigma (which includes the subjective experience of discrimination and the resulting alienation and social withdrawal), Stereotype Endorsement (the measure of agreement with negative stereotypes regarding people living with mental illnesses) and Stigma Resistance (the ability to counteract the internalization of stigma). The score of each separate factor was also included in the between-group comparisons.

Participants' real-world functioning was measured with the Global Assessment of Functioning (GAF) (56). The GAF is a frequently-used, simple and comprehensive instrument recommended in the DSM-IV-TR (57) for the assessment of social, occupational and psychosocial functioning; overall score ranges from 0 to 100, with higher scores representing better functioning.

The Subjective Well-Being Under Neuroleptic Treatment Scale short form (SWN-K) (58) is a measure assessing participant's self-perceived symptoms severity and level of functioning and providing an overall rating of subjective well-being. It is composed by 10 positive and 10 negative items, it is well-validated and it is one of the most used instrument to evaluate subjective well-being in people living with schizophrenia (59).

The Liverpool University Neuroleptic Side Effect Rating Scale (LUNSERS) (60) was used to assess the perceived impact of antipsychotic adverse effects. The LUNSERS is a 51-items scale addressing if and how frequently a subject experienced adverse effects in the last month. It includes a comprehensive list of antipsychotic-related adverse effects divided in 8 sub-scales (extrapyramidal side effects, psychic side effects, anticholinergic side effects, other autonomic reactions, allergic reactions, hormonal side effects, other reactions, “red-herrings”—items referring to symptoms that are not known as neuroleptic side-effects but useful to detect over-rating patients) and the total scores ranges from 0 to 164 for females and from 0 to 156 for males.

Finally, the Drug Attitude Inventory short-form (DAI-10) (61, 62) was used to assess participants' attitude toward prescribed medications.

### Statistical Analysis

Participants were divided in two groups on the basis of the PAUSS total score: subjects with a score ≥30 were allocated to the group “autistic schizophrenia” while those with a score <30 were allocated to the group “non-autistic schizophrenia.”

Parametric statistics were adopted regardless of the distribution of the data considering the size of the investigated sample and, given the exploratory nature of the investigation, in order to avoid type II errors (63, 64).

Between groups comparisons were performed using Pearson's Chi-squared or Fisher's Exact tests and *t*-test for categorical and continuous variables, respectively. For each investigate variable, Cohen's d was calculated as a measure of effect size. A d of 0.2 corresponds to a small effect size, a d of 0.5 corresponds to a moderate effect size and a d of 0.8 or higher corresponds to a large effect size (65). Additional confirmatory analyses were also conducted treating the PAUSS as a continuous measure using Pearson's r correlation.

Variables that emerged as significantly different in the between-groups comparisons were used as covariates in a backward logistic regression analysis to identify individual predictors of prominent ASD symptoms that could explain the largest portion of between-group variance.

Multicollinearity between individual predictors was assessed and was considered significant if the variance inflation factor (VIF) exceeded 4.0 (66). As the number of potential predictors introduced in the regression analysis was lower than one for every 10 observed subjects, which is recommended for logistic regressions according to conservative estimates (67, 68), the number of the included predictors was considered appropriate.

Statistical analyses were performed using SPSS version 15.0; *p*-values < 0.05 (two tailed) were considered significant.

## Results

### Sample Characteristics

A total of 94 subjects provided consent to participate and were included in the study. The sample was characterized by the presence of 21 (22.3%) female subjects and 73 (77.7%) male subjects. The mean age of the sample was 44.64 (SD ± 11.41) years. Thirteen (15.5%) participants showed prominent ASD symptoms and were included in the “autistic schizophrenia” group while 81 (74.5%) showed intermediate or low levels of ASD symptoms and were included in the “non-autistic schizophrenia” group.

### Between Groups Comparisons

As regards socio-demographic characteristics, significant between-group differences emerged regarding gender and education years: all participants showing prominent ASD symptoms were male (*p* = 0.036) and showed fewer education years (*p* < 0.001). No differences were observed regarding age (*p* = 0.177), age of onset (*p* = 0.118), antipsychotic medication dose (*p* = 0.387) and total medication dose (*p* = 0.251). More information regarding socio-demographic characteristics is reported in Table 1.

**Table 1:** Between-group comparison for socio-demographic characteristics.

**Variable**	**Total sample (*n* = 94) mean ±SD**	**Autistic Schizophrenia** **(PAUSS ≥30; *n* = 13)** **mean ±SD**	**Non-autistic Schizophrenia (PAUSS <30; *n* = 81) mean ±SD**	***t*-test/** **χ^2^-test**	***p-*value**	**Cohen's *d***
Age (years)	44.64 (±11.41)	48.61 (±7.62)	44.00 (±11.82)	−1.359	0.177	−0.406
Gender (M:F)	73:21	13:0	60:21	4.340	0.036^*^	-
Education (years)	10.29 (±3.10)	7.61 (±2.06)	10.71 (±3.03)	3.552	<0.001^**^	1.061
Age of onset (years)	25.34 (±7.56)	23.17 (±4.36)	25.66 (±7.90)	1.622	0.118	0.331
Antipsychotic medication dosage (DDD)	2.15 (±1.26)	2.43 (±1.61)	2.11 (±1.20)	−0.869	0.387	−0.254
Total medication dosage (DDD)	3.29 (±2.12)	3.92 (±2.20)	3.19 (±2.11)	−1.156	0.251	−0.344

*DDD, Defined Daily Dose; PAUSS, PANSS Autism Severity Score*.

* *p < 0.05;*

** *p < 0.01*.

Patients with prominent ASD symptoms showed both a more severe global clinical condition, as measured by the CGI-S score (*p* < 0.001) and more severe schizophrenia symptoms severity as measured by the PANSSminusPAUSS (*p* < 0.001).

They also showed worse overall real-world functioning, as measure by the GAF score, both at one-year and at one-week measurements (*p* < 0.001 for both).

No differences were observed regarding subjective well-being, as measured by the SWN-K (*p* = 0.853), antipsychotic-related adverse effects, as measured by the LUNSERS (*p* = 0.266) and attitude toward medications, as measured by the DAI-10 (*p* = 0.322). Between-groups comparisons for clinical and functional characteristics are reported in Table 2.

**Table 2:** Between-group comparison for clinical and functional characteristics.

**Variable**	**Total sample (*n* = 94) mean ±SD**	**Autistic Schizophrenia** **(PAUSS ≥30; *n* = 13)** **mean ±SD**	**Non-autistic Schizophrenia (PAUSS <30; *n* = 81) mean ±SD**	***t*-test**	***p-*value**	**Cohen's *d***
Global clinical severity (CGI-S)	3.80 (±1.10)	5.38 (±0.77)	3.58 (±1.00)	−6.127	<0.001^**^	−1.850
Non-autistic symptoms severity (PANSSminusPAUSS)	46.62 (±11.35)	61.54 (±13.70)	44.22 (±8.92)	−4.412	0.001^**^	−1.790
Real-world functioning, last year (GAF last year)	49.5 (±15.0)	32.92 (±10.19)	52.16 (±13.98)	4.753	<0.001^**^	1.420
Real-world functioning, last week (GAF last week)	52.60 (±12.7)	35.30 (±9.71)	55.40 (±10.77)	6.324	<0.001^**^	1.890
Subjective well-being (SWN-K)	78.80 (±17.6)	79.72 (±20.67)	78.65 (±17.26)	−0.186	0.853	−0.060
Medication-related adverse effects (LUNSERS)	12.40 (±10.80)	15.58 (±14.81)	11.96 (±10.07)	−1.120	0.266	−0.335
Attitude toward medications (DAI-10)	2.90 (±4.90)	1.70 (±6.00)	3.13 (±4.56)	0.996	0.322	0.300

*CGI-S, Clinical Global Impression-Severity; DAI-10, Drug Attitude Inventory; GAF, Global Assessment of Functioning; LUNSERS, Liverpool University Neuroleptic Side Effect Rating Scale; PANSS, Positive and Negative Syndrome Scale; PAUSS, PANSS Autism Severity Score; SWN-K, Subjective Well-Being Under Neuroleptic Treatment Scale short form*.

** *p < 0.01*.

No differences emerged regarding global internalized stigma experience, as measured by the ISMI total score (*p* = 0.773). However, considering the different factors of internalized stigma, participants with prominent ASD symptoms showed better stigma resistance (*p* = 0.022). No differences were observed regarding experiential stigma (*p* = 0.970) and stereotype enforcement (p = 0.679). More details on the comparison regarding internalized stigma are reported in Table 3.

**Table 3:** Between-group comparison for internalized stigma.

**Variable**	**Total sample (*n* = 94) mean ±SD**	**Autistic Schizophrenia** **(PAUSS ≥30; *n* = 13)** **mean ±SD**	**Non-autistic Schizophrenia (PAUSS <30; *n* = 81) mean ±SD**	***t*-test**	***p-*value**	**Cohen's *d***
Internalized stigma (ISMI)	62.95 (±11.62)	62.08 (±15.16)	63.09 (±11.06)	0.289	0.773	0.086
Experiential stigma (ISMI-Experiential)	36.09 (±8.79)	36.00 (±10.92)	36.10 (±8.48)	0.037	0.970	0.011
Stereotype endorsement (ISMI-Endorsement)	13.80 (±3.80)	14.15 (±3.62)	13.74 (±3.28)	−0.415	0.679	−0.123
Stigma resistance (ISMI-Resistance)	13.06 (±1.95)	11.92 (±2.32)	13.24 (±1.83)	366.00	0.022^*^	0.694

*ISMI, Internalized Stigma of Mental Illness; PAUSS, PANSS Autism Severity Score*.

* *p < 0.05*.

The supplementary analysis confirmed the observed results, as significant correlations emerged with the same variables that emerged as potential predictors in the previous analyses: the PAUSS total score was correlated with fewer years of education (*r* = −0.402, *p* < 0.001), greater global clinical severity (CGI-S, *r* = 0.798, *p* < 0.001) and non-autistic symptoms severity (PANSSminusPAUSS, r = 0.667, p < 0.001), and worse real-world functioning (GAF last year *r* = −0.712, *p* < 0.001 and GAF last week *r* = – 0.402, *p* < 0.001).

In particular, a significant correlation was observed for stigma resistance (ISMI-Resistance, *r* = −0.205, *p* = 0.048) but not for experiential stigma (ISMI-Experiential r = 0.120, p = 0.247) or stigma endorsement (ISMI-Endorsement *r* = 0.168, *p* = 0.105).

### Regression Analysis

Gender, years of education, global clinical severity, non-autistic schizophrenia symptoms severity, real-world functioning (both at the last-year and last-week assessments) and stigma resistance were introduced in the regression analysis as potential predictors. A more severe clinical condition, as measured by the CGI-S (*p* = 0.002), and fewer years of education (*p* = 0.015) emerged as individual predictors of prominent ASD symptoms. No significant collinearity emerged between individual predictors. Results of the logistic regression analysis are reported in Table 4.

**Table 4:** Individual predictors of autistic schizophrenia.

**Dependent variable**	**Individual predictors**	**B**	**Exp (B)**	**VIF**	***p-*value**
Autistic Schizophrenia (PAUSS ≥ 30)	Global clinical severity (CGI-S)	2.735	15.407	1.058	0.002^**^
	Education (years)	−0.545	0.580	1.058	0.015^*^
	*Model: Chi^2^= 43.249*				<0.001^**^
	*Cox-Snell R^2^ = 0.369*,				
	*Nagelkerke R^2^ = 0.668*				

*Potential predictors: Gender, Education (years), Global clinical severity (CGI-S), Non-autistic symptoms severity (PANSSminusPAUSS), Real-world functioning, last year (GAF last year), Real-world functioning, last week (GAF last week), Stigma resistance (ISMI-Resistance)*.

*CGI-S, Clinical Global Impression-Severity; GAF, Global Assessment of Functioning; ISMI, Internalized Stigma of Mental Illness; PANSS, Positive and Negative Syndrome Scale; PAUSS, PANSS Autism Severity Score*.

* *p < 0.05;*

** *p < 0.01*.

## Discussion

Participants with prominent ASD symptoms represented a minority of the total included sample and showed various distinctive features: for instance, they were all of male gender, which was an expected finding considering the higher prevalence of ASD features in male subjects (69–71).

Of note, participants in the “Autistic schizophrenia” group showed greater functional impairment, both at the one-year and at the one-week assessment of the GAF: this result confirms the negative impact of ASD symptoms on functional outcomes of people living with schizophrenia, a fact already attested by studies conducted on larger samples of participants (19–21).

Global symptoms severity, as well as the severity of non-autistic schizophrenia symptoms, was greater in participants with higher levels of ASD symptoms.

These subjects also showed fewer years of education. To some extent, this can be considered a potential indirect proxy of worse cognitive performance; however, this could also represent a marker of worse general adversity, including other important factors such as potential cognitive difficulties, psychological trauma—including bullying, less supportive home environment, and worse economic status.

Global symptoms severity and years of education also emerged as individual predictors of prominent ASD symptoms, explaining the largest portion of variance between the two groups.

Taken together, these findings confirm that people living with schizophrenia showing prominent ASD features are characterized by a more problematic and severe disorder, with worse cognitive, clinical and functional outcomes (27, 42).

Despite presenting greater functional impairment and a more severe clinical condition, subjects with prominent ASD symptoms, compared to other participants, did not show worse subjective well-being or worse overall internalized stigma experience. In fact, not only comprehensive self-stigma severity was not greater in participants with more severe ASD symptoms, but these subjects showed better stigma resistance, as attested by a lower score in the ISMI Resistance factor.

This is an interesting finding which could have different possible explanations. People diagnosed with schizophrenia showing prominent ASD features present substantial impairments in the ability to appropriately evaluate the quality of everyday functioning (22): this issue could consistently mitigate the impact of worse functional outcomes on subjective well-being but also, to a lesser extent, to reduce the internalization of stigmatizing experiences related to functional impairment. Deficits in social cognition (20, 51, 72) could also have a protective role in the internalization of stigma, as a limited understanding of social cues and social contexts could enhance the resistance to internalization of stigma experiences.

Individuals with prominent ASD features appear to adopt different coping mechanisms, compared to other people living with schizophrenia (26), and it is possible that this coping profile could contribute to a better stigma resistance. Finally, these subjects also present better real-world social acceptability (27), which could determine a lower exposure to social discrimination and stigma-related events: this hypothesis, however, has less empirical support, as no between-group difference was observed in the Experiential stigma factor of the ISMI.

Findings of the present study are in contrast with the result of a previous paper investigating this topic, which reported that ASD features represent a predictor of worse internalized stigma (41). This discrepancy can be due to important methodological differences between the studies: the study by Baron-Cohen et al. evaluated ASD features with the Autism Spectrum Quotient (73), a widely used and well-validated measures of ASD features that, however, relies of self-assessment, while in the present work ASD symptom were evaluated using a validated instrument devised specifically to assess ASD features in people living with schizophrenia and based on the rating of trained clinicians. Moreover, autistic features were analyzed as a continuous measure in the study by Bechi et al. while the present work focused on assessing individuals characterized by high levels of ASD symptoms. It is also possible that autistic features could have different correlates depending on their severity but also on the severity of schizophrenia symptoms (29).

These findings are of considerable clinical relevance: as individuals with prominent ASD features appear to be characterized by specific features, they could present substantial differences in the response to specific treatments. In fact, recent evidence suggests that ASD symptoms appear to be associated with poorer response to antipsychotic treatment in schizophrenia (24, 25), a finding that could be indirectly related to the greater symptoms severity observed in “Autistic schizophrenia” participants. These patients, however, could represent valid candidates to targeted psychosocial interventions: for instance, cognitive remediation appears to produce greater cognitive and functional gains in participants that are more clinically compromised (74, 75). These implications also highlight the importance of assessing ASD symptoms in people living with schizophrenia into real-world, everyday clinical practice.

This study has some noticeable points of strength. To the best of our knowledge, this represents the first assessment of internalized stigma and of subjective well-being in people living with schizophrenia showing prominent ASD features, comprising also a comparison with participants with lower levels of ASD symptoms and an assessment of different clinical and functional features.

The recruitment of participants in a clinical rehabilitation context without restrictive inclusion criteria increases the generalizability of the results to real-world, day-to-day clinical reality, while the use of simple and widely used assessment tools allows to easily reproduce the result of the study.

Finally, the inclusion of participants with a well-defined diagnosis and the clinician-rated assessment of ASD symptoms contribute to the validity of the findings.

The present work has some limitations. The study has a cross-sectional design, which does not allow to determine a direction of causality in the associations observed in the analyses. The lack of a direct assessment of neurocognitive and socio-cognitive performance and of separate areas of real-world functioning of participants also represent a potential limitation; however, employing a complex and detailed assessment of cognition and functioning would have not been representative of the real-world context of rehabilitation practice. The size of the recruited sample was relatively small, and this could lead to an increased risk of type II errors (false negatives); however, the sample size was sufficient to observe significant differences in some of the key investigated aspects. Effect sizes were also minimal-to-small in variables that did not emerge as significant in the analyses, confirming the solidity of the results.

Finally, ASD symptoms measured with the PAUSS may have some measure of overlap with negative symptoms, as many items of the instrument belong to the negative symptoms scale of the PANSS.

This issue represents a consistent limitation of the present study.

In fact, limited social interactions, social apathy and lack of spontaneity can be considered core characteristics of both negative and autistic symptoms. However, important differences exist between the two domains: emotional withdrawal and diminished, or “flattened,” affectivity represent a truly essential aspect of negative symptoms (14) and cannot be considered an autistic characteristic. On the contrary, mannerisms and autistic preoccupations, as well as impairment in abstract thinking, are typical autistic features that are not associated with the negative symptoms dimension (5, 76).

In fact, the validity and the accuracy of the PAUSS to assess autistic features was validated in previous studies comparing it to gold-standard assessment interviews (48, 77) and its specificity has been attested in very large samples of participants (46), also taking into account the severity of schizophrenia symptoms (22, 27, 51). Moreover, while the negative subscale of the PANSS has been defined a priori, several models of factor analysis have provided different and highly consistent negative symptoms factors that have considerably lower levels of overlap with the PAUSS (76, 78, 79).

Further understanding the differences and the specificity of correlates of negative symptoms and ASD features measured with the PAUSS in people living with schizophrenia spectrum disorders represents an interesting perspective for future research. In particular, this could be appropriately performed by evaluating the severity of negative symptoms with one of the so-called “second generation” scales, such as the Brief Negative Symptom Scale (80), for different reasons. On one hand, this would allow to avoid collinearity issues between the PAUSS and the negative symptoms subscale or negative symptoms factor of the PANSS that could arise by introducing both domains in covariates in generalized linear models and lead to a high risk of consistent type II errors (81); on the other, the PANSS does not currently represent the best instrument to assess negative symptoms, according to recent guidance, while “second generation” scales are more accurate and appropriate (14).

In conclusion, people living with schizophrenia with prominent ASD symptoms do not appear to show worse internalized stigma or worse subjective well-being despite having greater functional impairment and a more severe clinical presentation; on the contrary, they show better stigma resistance. Therefore, it is possible that ASD symptoms could have a protective role in the internalization of stigma. Future studies should focus on further investigating the correlates of ASD features in different contexts and with larger samples of participants and to better understand the impact of ASD symptoms on the effectiveness of specific pharmacological and psychosocial interventions.

## Data Availability Statement

The raw data supporting the conclusions of this article will be made available by the authors, without undue reservation.

## Ethics Statement

The studies involving human participants were reviewed and approved by Ethics Committee of ASST Spedali Civili of Brescia. The patients/participants provided their written informed consent to participate in this study.

## Author Contributions

SB, GN, and AV: conceptualization, methodology, writing, review and editing. SB, GN, DM, and AV: data curation. GN and DM: formal analysis. PCa, PCo, AM, VA, VR, and JL: investigation. SB, GD, CT, PV, and AV: supervision. All authors contributed to the article and approved the submitted version.

## Conflict of Interest

The authors declare that the research was conducted in the absence of any commercial or financial relationships that could be construed as a potential conflict of interest.

## Publisher's Note

All claims expressed in this article are solely those of the authors and do not necessarily represent those of their affiliated organizations, or those of the publisher, the editors and the reviewers. Any product that may be evaluated in this article, or claim that may be made by its manufacturer, is not guaranteed or endorsed by the publisher.
